# Case report: Efficacy of immunotherapy as conversion therapy in dMMR/MSI-H colorectal cancer: a case series and review of the literature

**DOI:** 10.3389/fimmu.2024.1352262

**Published:** 2024-02-01

**Authors:** María San-Román-Gil, Iñigo Martínez-Delfrade, Víctor Albarrán-Fernández, Patricia Guerrero-Serrano, Javier Pozas-Pérez, Jesús Chamorro-Pérez, Diana Rosero-Rodríguez, Pilar Sotoca-Rubio, Ana Maria Barrill-Corpa, Víctor Alia-Navarro, Carlos González-Merino, Coral García-de-Quevedo-Suero, Victoria López, Ignacio Ruz-Caracuel, Cristian Perna-Monroy, Reyes Ferreiro-Monteagudo

**Affiliations:** ^1^ Medical Oncology Department, Hospital Universitario Ramon y Cajal, Madrid, Spain; ^2^ Medical Oncology Department, Royal Marsden Hospital, London, United Kingdom; ^3^ Pathology Department, Hospital Universitario Ramon y Cajal, Madrid, Spain

**Keywords:** immunotherapy, neoadjuvant, colorectal cancer, microsatellite instability, oligometastatic, conversion therapy

## Abstract

Immunotherapy has demonstrated a role in the therapeutic landscape of a small subset of patients with colorectal carcinoma (CRC) that harbor a microsatellite instability (MSI-H) status due to a deficient DNA mismatch repair (dMMR) system. The remarkable responses to immune checkpoint inhibitors (ICIs) are now being tested in the neoadjuvant setting in localized CRC, where the dMMR/MSI-H status can be found in up to 15% of patients, with remarkable results obtained in NICHE2 and 3 trials, among others. This case series aims to report our experience at a tertiary center and provide a comprehensive analysis of the possible questions and challenges to overcome if ICIs were established as standard of care in a neoadjuvant setting, as well as the potential role they may have as conversion therapy not only in locoregional advanced CRC but also in oligometastatic disease.

## 1 Introduction

Colorectal cancer (CRC) is the third most common malignancy diagnosed worldwide and the second in terms of cancer-related mortality. Approximately 20% of patients are metastatic at diagnosis, while half of the initially localized ones will develop metastases throughout the disease. Most of these patients will be treated with a palliative purpose, with a median overall survival of 30 months (1).

For several years, the standard of care (SOC) for unresectable locally advanced or oligometastatic (resectable or potentially resectable) CRC has been based on a combination of 5-Fluorouracil (5-FU)-based chemotherapy with or without an anti-vascular endothelial growth factor (VEGF) antibody when *RAS* or *BRAF* were mutated, or an anti-epidermal growth factor receptor (EGFR) agent, when they were found in the wild-type status. Therefore, nowadays, the detection of *KRAS*, *NRAS* and *BRAF* mutations as well as the deficit of mismatch repair proteins (dMMR) and subsequent high microsatellite instability (MSI-H) testing are compulsory in order to establish not only the best treatment sequence but also the prognosis of the disease. Depending on the response, surgery was performed after several cycles, and the same chemotherapy scheme was considered to complete 6 months-treatment between pre and post-surgery (2).

However, as it happened with metastatic CRC, this precept may change when the dMMR/MSI-H status is identified since this status is a trustworthy biomarker of long-term response to immune checkpoint inhibitors (ICIs) targeting programmed death-1/programmed death ligand-1 (PD-1/PD-L1) and cytotoxic T lymphocyte antigen- 4 (CTLA-4); therefore, the use of ICIs has become the SOC in this subgroup of patients in the metastatic first-line setting. The dMMR/MSI-H status is harbored by 5-15% of all CRC patients, with a higher incidence in early-stage CRC (3). Besides, the identification of dMMR/MSI-H tumors can assist in the necessity of genetic counselling for Lynch Syndrome (LS). However, 70-85% of these dMMR/MSI-H tumors are formed sporadically, mostly by the hypermethylation of the *MLH1* promoter. It can take place concurrently with a *BRAF* V600E mutation and may or may not be associated with the loss of PMS2 (4, 5). Only 1 to 3% of CRC corresponds to LS, which is caused by a germline mutation in the MMR genes (*MLH1, MSH2, MSH6* or *PMS2*) or in the epithelial cell adhesion molecule (EPCAM), which provokes an epigenetic silencing of *MSH2* (5).

The family of MMR proteins coded by MMR genes oversee the repairing of insertions and deletions formed during DNA replication, especially in the microsatellite regions of DNA, which consist of repeated sequences of 1-6 nucleotides located throughout the DNA, especially near the coding regions. Therefore, the accumulation of errors in those regions leads to the mentioned MSI-H, which is associated with an increased mutational rate that can be up to 100-fold greater (>12 mutations per 10^6^ DNA bases) than proficient MMR, microsatellite-stable tumors or low microsatellite instability (pMMR/MSS-MSI-L; <8.24 mutations per 10^6^ DNA bases) (6, 7).

The identification of the dMMR and MSI-H status should be done by using an immunohistochemistry (IHC) assay and a multiplex polymerase chain reaction (PCR) test along with capillary electrophoresis, respectively; it is highly recommendable to use both methods, since using only one assay may be misjudging (8). Besides, both MSI and dMMR can also be assessed through next-generation sequencing (NGS) techniques but they are not available universally (6). Even though the terms of dMMR and MSI-H are usually associated, some exceptions may be found since the mutation of one of the MMR, such as MSH6 or PMS2, may reach an MSS or MSI-L status and not meet the criteria for MSI-H and, much more infrequently, the other way around can be seen (pMMR status with MSI-H) (9).

Therefore, the rationale for the use of immunotherapy in dMMR/MSI-H CRC is based on the vast amount of neoantigens that these tumors can produce and present as peptides through major histocompatibility complex (MHC) class I molecules on their surfaces. These peptides are recognized by T cell receptors (TCR), but this interaction is not enough to launch an immune response against tumoral cells since this TCR-MHC complex is modulated by co-stimulatory and co-inhibitory signals. These checkpoint inhibitors may be blocked using ICIs which can target either the co-inhibitory receptors PD-1 or CTLA-4, located on the surface of T-cells and other immune cells, or their ligands, such as PDL-1, which are presented on tumor cells and other immune cells. Therefore, by using ICIs the escape mechanism to the immune system of tumoral cells is revoked and T-cell response is enhanced against malignant cells (10). Besides, these tumors are known as immunogenic since their tumor microenvironment is heavily infiltrated by CD8+ tumor-infiltrating lymphocytes (TILs), CD4+ T helper 1 TILs and macrophages in their M1 status, as well as other antigen-presenting cells. The number of TILs is comparatively higher in MSS tumors and may also be even higher than the number of neoplastic cells but there is also an upregulation of the expression of the co-inhibitory signalization through CTLA-4, PD-1, PD-L1, LAG-3, and IDO that support the immune evasion. However, the PD-L1 expression in CRC is strikingly scarce in tumoral cells, being expressed on myeloid cells surrounding and, therefore, it is not a reliable predictive biomarker of response (11–14).

The role of immunotherapy in the neoadjuvant setting still lacks randomized trials to be completely defined, although some light has been shed on this field in the last few years. Up to now, the role of neoadjuvant chemotherapy in locally advanced tumors has not been proven successful in general, especially in MMR–deficient tumors, and surgery keeps being the standard at these stages, except for the unresectable cases which must be treated as metastatic with or without an anti-VEGF or anti-EGFR agent, depending on the mutational status and resectability. In the phase II/III FOxTROT trial, the use of 3 cycles of preoperative oxaliplatin-fluoropyrimidine chemotherapy showed a clear benefit in down-staging the locally advanced tumors, with fewer incomplete resections, and a better 2-year disease control. However dMMR tumors experimented a statistically lower response to chemotherapy than pMMR tumors and 70% of these patients experiencing no regression (15). The recent data from clinical trials using ICIs on this subgroup of patients (16–20) and case reports like the ones we are presenting point out the role of immunotherapy in the neoadjuvant setting. However, this is a small case series and, therefore, in order to validate our findings, larger patient cohorts and phase III clinical trials are required.

## 2 Cases description

Here we present our four case reports of two unresectable locally advanced CRC and two oligometastatic CRC, including a patient with non-locoregional nodal disease and another one with a large unique liver relapse (oligometastatic disease). They all experienced great responses to pembrolizumab in monotherapy. Several cases of pathological complete response (pCR) or major PR (MPR) following immunotherapy have been described in the literature but, up to our knowledge, there have been no published reports of pCR or MPR in oligometastatic disease. Moreover, genetic profiling was performed at our centre with potentially targetable molecular alterations found which could be useful if a relapse occurs. The characteristics of the four case reports are summarized in **Table 1**.

**Table 1:** Clinical characteristics of the 4 case reports presented.

	Case Report 1	Case Report 2	Case Report 3	Case Report 4
**Family history** **Tumor characteristics**	Yes MLH-1 and MSH-2 deficient poorly differentiated adenocarcinoma - Grade 3	No MLH-1 and PMS-2 deficient moderately differentiated adenocarcinoma - Grade 2	No MLH-1 and PMS-2 deficient adenocarcinoma with a component of signet ring cell -Grade 3	No MLH-1 and PMS-2 deficient well-differentiated adenocarcinoma -Grade 1
**Molecular findings^**	dMMR *RAS/BRAF* wild-type status (NGS not performed)	dMMR *BRAF* V600E/D (NGS performed)	dMMR *RAS/BRAF* wild-type status (NGS: *RET::NCOA4*)	dMMR *BRAF* V600E/D (NGS performed)
**Staging pre-ICI**	cT4N2Mx (pancreatic and liver infiltration)	cT3-T4N2M0 (Locally advanced unresectable)	cT4N2M1 (non-locoregional nodes and uterine and adnexal infiltration)	cTxNxM1 (liver relapse)
**Number of cycles before surgery**	5 (pembrolizumab)*	5 (pembrolizumab)*	9 (pembrolizumab)*	11 (pembrolizumab)*
**Clinical Response**	cT3N1-2Mx (no signs of pancreatic and segment IV liver infiltration)	cT2N1Mx (remarkable response of the primary mass and locoregional nodal disease)	cT3N1M1	cTxNxM1 (liver relapse - major partial response)
**Pathological Response**	pCR	pCR	MPR [ypT3 ypN1a (1/27)]	pCR
**Germline testing^**	Yes – sporadic (h*MLH1*)	No (*BRAF*m + h*MLH1*)	Yes – sporadic (h*MLH1*)	Yes – heterozygous p.G396D *MUTYH* ^γ^

ICI: immune-checkpoint inhibitors. pCR: Pathological Complete Response. MPR: Major Pathological Response. hMLH1: hypermethylation of MLH1 promoter.

^Molecular findings were obtained using a PCR method for KRAS and NRAS-BRAF mutation (Idylla™) and an NGS gene panel for further characterization (Oncomine™ Focus Assay), which enables the detection of variants in 52 genes relevant to solid tumors, such as relevant hotspots, single nucleotide variants (SNVs), indels, copy number variations (CNVs), and gene fusions. The dMMR status was determined in the first tissue available (endoscopic biopsy) by using an IHC test. MLH1 promotor hypermethylation was assessed by using a methylation-specific PCR.

*Pembrolizumab posology was every three weeks in the neoadjuvant setting. Case Report 3 continued with adjuvant pembrolizumab every six weeks.

^γ^This mutation has not been linked to a higher risk of CRC.

### 2.1 Case report 1

A 76-year-old woman with a personal medical history of high blood pressure (HBP), hypercholesterolemia, and diabetes, was referred after positive results in a fecal occult blood test. No constitutional syndrome was described. Regarding family history, she reported a brother diagnosed with colon cancer who died at 60 years of age and a sister diagnosed with ovarian cancer at 66 years, who is alive. A colonoscopy found a stenosing mass at the hepatic flexure, and biopsies were performed that revealed a dMMR (MLH-1 and MSH-2 deficient) poorly differentiated adenocarcinoma. The molecular study revealed a RAS/BRAF wild-type status Suspicious adjacent lymph nodes, but no distant metastases were found on the staging CT scan, and serum carcinoembryonic antigen (CEA) was within the normal levels (<5 ng/mL). Therefore, the patient was scheduled for surgery, but during the surgical procedure, pancreatic and segment IV liver infiltration of the colonic mass was observed, and the patient underwent a palliative ileocolic bypass to avoid clinical obstruction.

With the diagnosis of unresectable locally advanced dMMR/MSI-H CRC due to locoregional infiltration of surrounding organs (cT4N2Mx), the patient was referred to Medical Oncology. Based on the results of the KEYNOTE 177 trial, pembrolizumab was initiated in June 2021. After 4 cycles, the interval CT scan showed a remarkable response of the primary mass with no longer evidence of pancreatic or liver infiltration (cT3N1-2Mx; **Figure 1.1**). A 5**^th^** cycle was administered before she underwent an extended right hemicolectomy in September 2021. No residual tumor foci were identified macroscopically or microscopically, consistent with a pathological complete response (pCR; ypT0, ypN0 [0/58]) to neoadjuvant treatment (Modified Ryan scoring of 0) (**Figure 1.2**).

**Figure 1:**
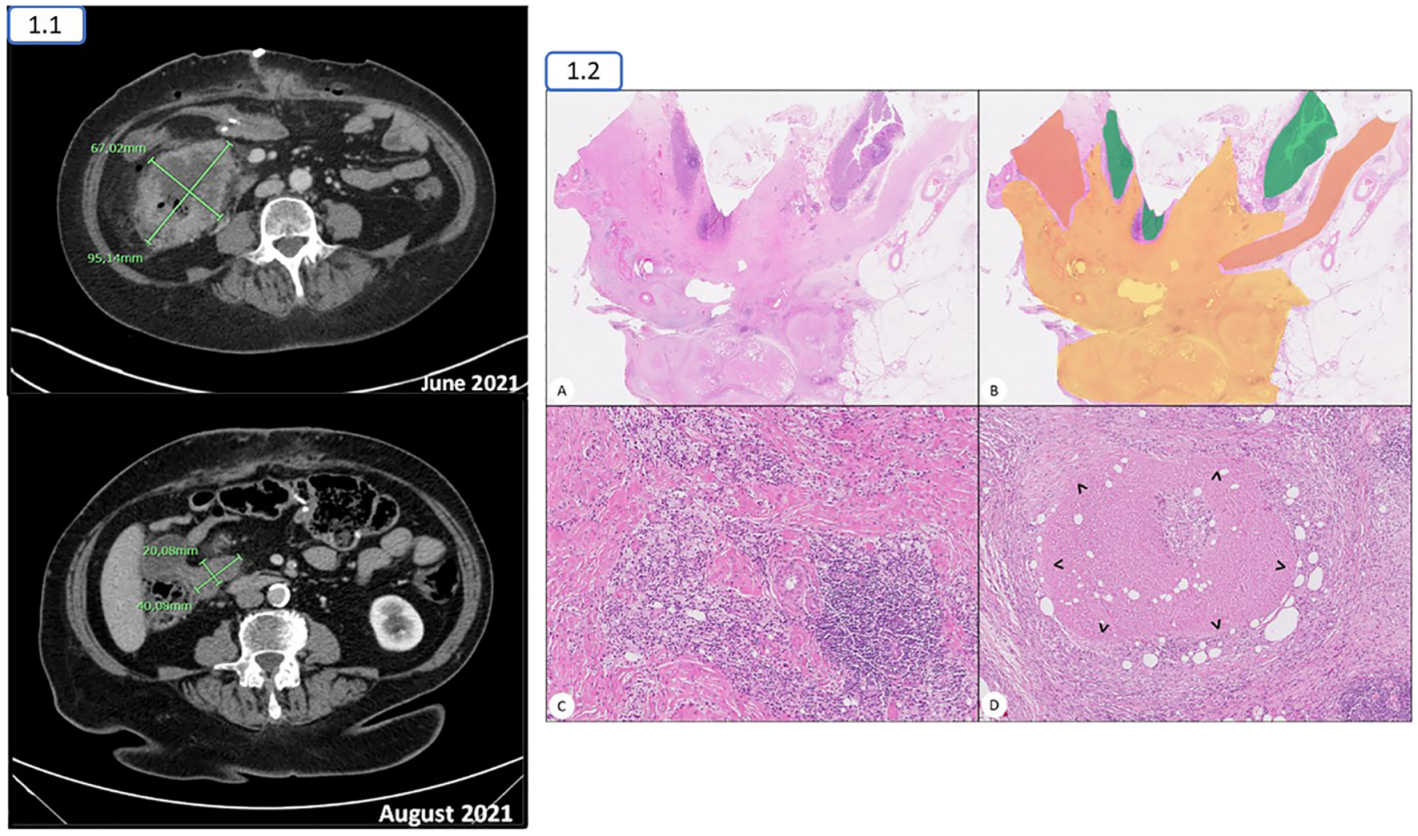
1.1. The right colonic mass experienced a considerable decrease in its size from June to August 2021, with no longer evidence of liver or pancreatic infiltration 1.2. **(A, B)** Transmural full-thickness section of the rectum showing regression changes with no residual neoplasia. Normal mucosa and muscularis propia are coloured green and orange, respectively. The area showing regression changes is coloured in yellow. **(C)** Some areas of regression showed a mixture of lymphoid and macrophage infiltrates admixed in a collagenous residual stroma. **(D)** Numerous necrotizing granulomas with a rim of macrophages (>) were present.

Since no evidence of administering adjuvant therapy when achieving a pCR has been assessed in clinical trials, the case was discussed at MDT, and she has continued follow-up with no evidence of relapse. Considering her family history and the absence of BRAF mutated colon, germinal genetic testing was performed based on positive Bethesda criteria, with no pathogenic findings. NGS tissue testing could not be done since there were no tumoral cells in the surgical specimen (pCR), and the biopsy obtained from colonoscopy had been used in previous studies so there was insufficient tumoral sample to analyze. The hypermethylation of the *MLH1* promoter was confirmed by using a PCR method.

### 2.2 Case report 2

A 62-year-old man with a medical history of HBP and right retinal vein occlusion debuted with a 2-month duration of sporadic abdominal pain with no constitutional syndrome or other symptomatology. No family history to report. He had an abdominal echography performed by his general practitioner, which revealed a mass at the hepatic flexure of the colon, and the diagnostic study was initiated. No evidence of distant disease was found with a staging body CT scan, but there was extensive locoregional infiltration (cT3-T4N2bM0), and colonoscopy found a mildly stenosing mass at the hepatic flexure. Biopsies were performed that revealed a dMMR (MLH-1 and PMS-2 deficient) adenocarcinoma. The molecular study revealed a *BRAF* V600E/D mutation. The CEA was within normal levels. Due to the locoregional extension, no surgical procedure could be performed, and the MDT decision was to initiate systemic treatment.

**Figure 2:**
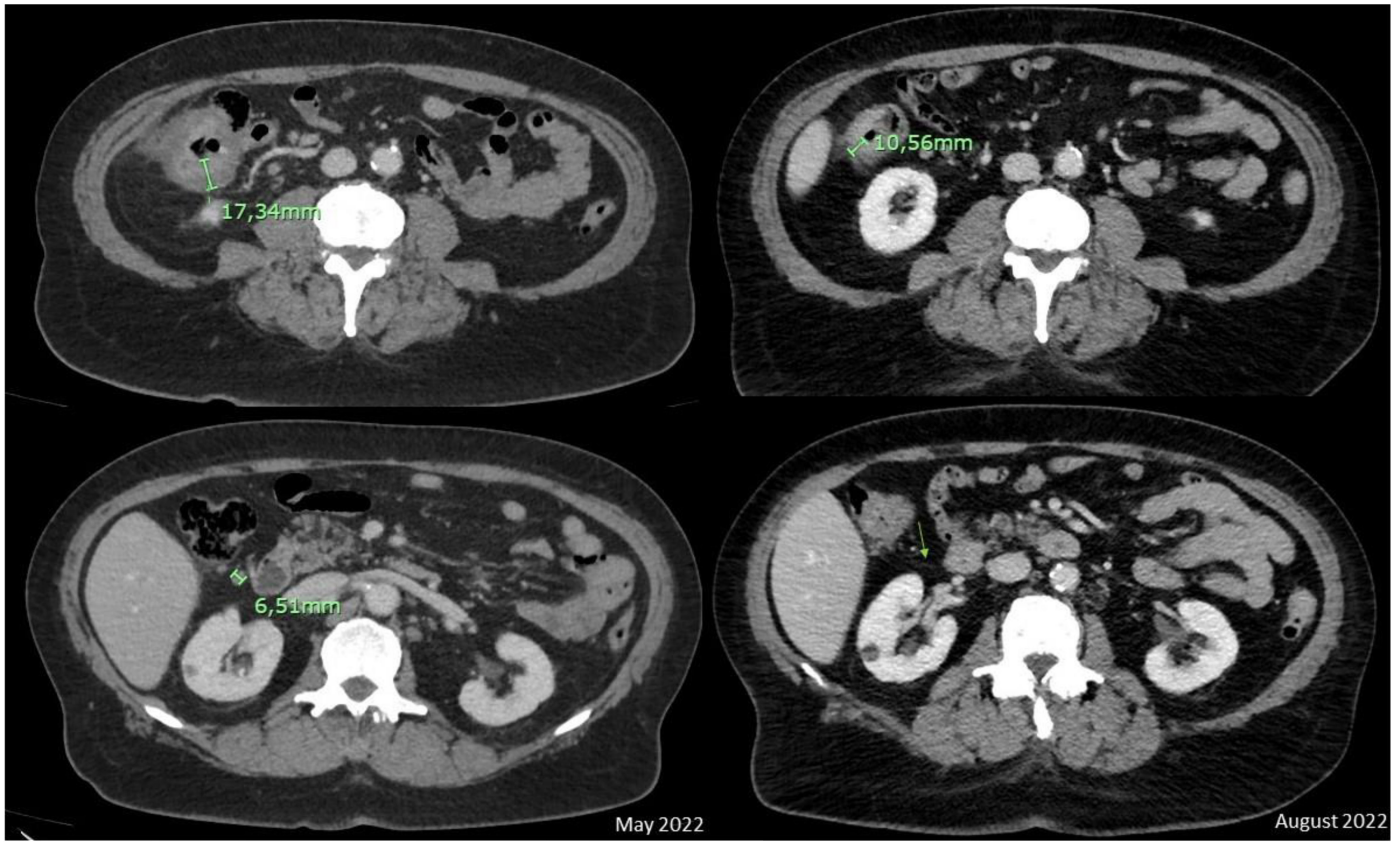
A major radiologic response was observed with a clear reduction of the colonic neoplasm and no longer evidence of adjacent suspicious nodes between the basal CT scan and interval CT scan in August 2022. The green arrow represents the absence of lymph node after 5 cycles of pembrolizumab.

With the diagnosis of unresectable locally advanced dMMR/MSI-H CRC, the patient was referred to Medical Oncology, and based on the results of the KEYNOTE 177 trial, pembrolizumab was initiated in June 2022. After 4 cycles, the interval CT scan showed a remarkable response of the primary mass and locoregional nodal disease (cT2N1Mx; **Figure 2**). A 5th cycle was administered before surgery in September 2022. As happened with the first reported case, a pathological complete response (pCR) was reported (ypT0, ypN0 [0/43]) to neoadjuvant treatment (Modified Ryan scoring of 0).

The patient also continued follow-up with no evidence of relapse. No germline genetic testing was indicated due to the *BRAF* V600E/D mutation found and the absence of a family history. The NGS panel test revealed the same mutation as the molecular study [*BRAF* c.1799T>A p.Val600Glu], with no concurrent drivers, and hypermethylation of the *MLH1* promoter was found by PCR.

### 2.3 Case report 3

A 56-year-old woman with a personal history of endometriosis debuted with a three-month history of slight constipation and abdominal pain. No family history to report. Active smoker since puberty, no other toxic habits to declare. She went to the emergency department due to an episode of uncontrolled hypogastric pain and the blood tests performed revealed elevated acute reactants; therefore, an urgent abdominal-pelvic CT scan was performed to rule out an acute event, which found a stenosing neoplasm at the sigma with retroperitoneal and iliac pathological nodes, uterine and adnexal infiltration, and suspicious metastatic liver nodes. She was admitted to the hospital to complete the diagnosis. A colonoscopy found a completely stenosing mass (100%) at the sigma-recto union, although no clinical obstruction was observed. Biopsy revealed a dMMR (MLH-1 and PMS-2 deficient) adenocarcinoma with a component of signet ring cell, BRAF/RAS wild type. CEA was slightly elevated (5.1 ng/mL [laboratory values: normal value <5 ng/mL, 5-10 ng/mL could be due to tobacco or another abdominal inflammatory process]). An MRI was done to rule out metastatic liver disease, revealing that the lesions were compatible with liver haemangiomas. Therefore, with the diagnosis of metastatic dMMR/MSI-H CRC (due to non-locoregional nodes and uterine and adnexal infiltration), the patient was referred to Medical Oncology, and pembrolizumab was initiated in August 2022 based on KEYNOTE 177 data.

The interval CT scan after 5 cycles showed a remarkable response and CEA within normal levels, although there were radiological findings compatible with pyometra or a right-annex abscess (**Figure 3.1**). The patient was asymptomatic except for slight abdominal pain controlled with non-opioid analgesics. A gynaecological exploration with biopsy collection was performed, confirming an inflammatory process with no neoplastic cells, and microbiological cultures yielded results of *Streptococcus intermedius* and *Peptoniphilus species*. Oral antibiotic therapy was initiated, and follow-up was recommended. However, the collection continued to grow considerably in the following weeks despite antibiotic therapy, and a grade III right ureter hydronephrosis was found on a CT scan after the ninth cycle. Therefore, she was proposed for pelvic collection drainage, which was performed in April 2023 with polymicrobial findings (*Enterococcus faecium, Streptococcus constellatus*, and *Streptococcus intermedius*) and no evidence of underlying neoplasm after the pathological examination.

**Figure 3:**
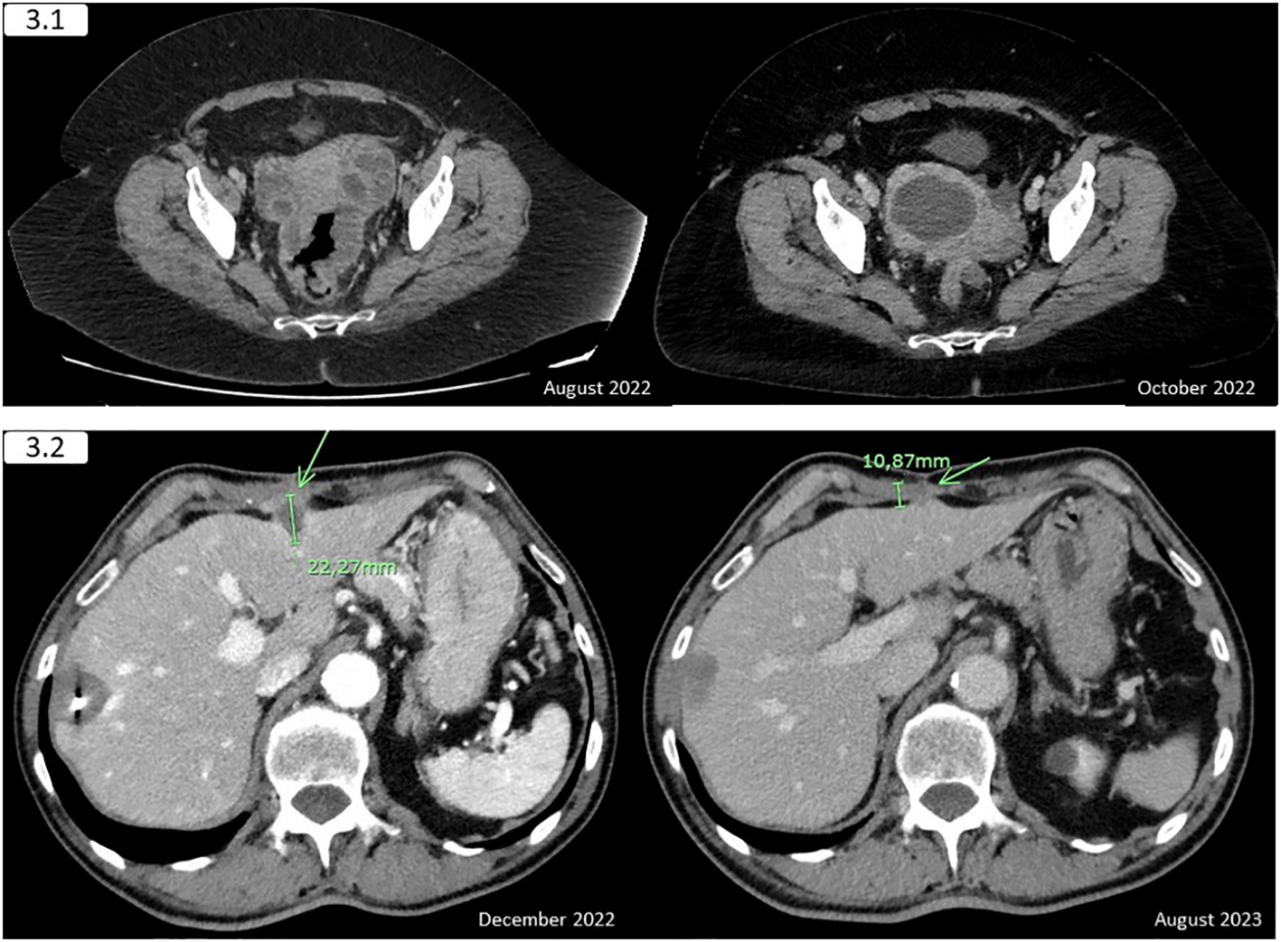
3.1 (Case report 3) - The interval CT scan revealed an amelioration of the rectal and adnexal infiltration, with the apparition of a pyometra or a right-annex abscess that provokes an ipsilateral ureterohydronephrosis. A decrease in the abdominopelvic nodes was also observed. 3.2 (Case report 4) - The previously treated with radiofrequency 33-millimetre liver lesion in the right hepatic lobe showed no changes and a remarkable decrease is appreciated in the segment III metastasis, remaining the signs of the hepatic capsule and anterior abdominal wall infiltration. The green arrow indicates the location of the infiltrating liver lesion.

Due to the gastrointestinal microorganisms obtained in both cultures, suspicion of a fistula from the gastrointestinal tract was raised, and an MRI confirmed it. Therefore, due to the significant response of the primary tumor and nodal disease, the case was proposed for radical surgery at the MDT. A resection of the primary tumor plus extended lymphadenectomy, hysterectomy, and double adnexectomy was performed in June 2023. The pathological report indicated a major pathological response (mPR; less than 10% of viable tumoral cells in the specimen) ypT3 ypN1a (1/27). After MDT discussion, pembrolizumab was restarted in August 2023 to complete two years of pembrolizumab, as it was stage IV before surgery, and the KEYNOTE 177 protocol allowed continued treatment for up to two years after surgery (although we have no published data from these patients since it was censored).

The NGS panel test performed on the surgical specimen revealed a *RET::NCOA4* fusion, confirmed by FISH, with no other molecular alterations found among the genes targeted, and no evidence of relapse has been proven since. Besides, the germline genetic testing revealed no pathogenic findings and the hypermethylation of the *MLH1* promoter resulted in positive.

### 2.4 Case report 4

A 74-year-old man with no personal or family history to report was admitted to the hospital in August 2020 due to 7-day rectal bleeding along with melena. No constitutional syndrome or abdominal pain was reported. He was diagnosed with an active duodenal ulcer and left colon neoplasm by performing an upper endoscopy and colonoscopy, respectively, during his admission at the hospital. The pathological report of the biopsy revealed a dMMR (MLH-1 and PMS-2 deficient) adenocarcinoma, with a *BRAF* V600E mutation. The CEA was within normal levels. A staging CT scan revealed two liver lesions in the VII segment, undetermined by this technique. An MRI confirmed the metastatic nature of these lesions and, after discussion at the MDT reunion, a systemic neoadjuvant treatment based on 5-FU chemotherapy was initiated in October 2020 (FOLFOX) after labelling liver metastases. DPYD testing shown heterozygous variants with no known clinical relevance [c.85T>C (p.Cys29Arg, rs1801265), c.1218G>A (p. Met406Ile, rs61622928) and c.2343T>A (p.Ile781Asn)]. Therefore, full doses were established. However, after the second cycle, the patient was admitted to the intensive care unit due to abdominal sepsis because of neutropenic typhlitis along with an acute bilateral pulmonary thromboembolism.

After several weeks, he was discharged from hospital. However, after this episode, neoadjuvant treatment was stopped and he was elected for a left hemicolectomy that took place in December 2020, with a pathological stage IIIB in the surgical specimen (pT3pN1a [1/24]). Post-surgical CT scan revealed no further disease, and radiofrequency ablation could be done on the two liver metastatic lesions in February 2021. Although the patient was recommended to continue chemotherapy to complete 6 months of treatment, he refused it and started follow-up.

In January 2023, a 22-millimeter lesion appeared in liver segment II-III, breaking Glisson’s capsule and infiltrating the abdominal wall on the CT scan. The patient required opioid treatment due to the pain associated with that lesion. At the MDT meeting, the decision was to initiate systemic treatment due to the local extension of the lesion and reevaluate for considering surgery.

Based on the results of the KEYNOTE 016 trial, although only 2 cycles of FOLFOX were administered three years before, pembrolizumab was initiated in February 2023. The first restaging scan showed a remarkable partial response, and the patient was proposed for surgery, but he refused and preferred to continue with immunotherapy. However, after eight cycles the second restaging scan showed an even better response (**Figure 3.2**), and the patient was willing to undergo surgery, which was performed in October 2023. He received three additional cycles before going through surgery.

Therefore, surgery consisted of a laparoscopic segmentectomy, and the pathology report revealed no residual tumor foci identified macroscopically or microscopically, consistent with pathological complete response (pCR; ypT0) to neoadjuvant treatment (Modified Ryan scoring of 0).

Since no evidence of administering adjuvant therapy when achieving a pCR has been assessed in clinical trials, the case was discussed at MDT and with the patient, with the mutual decision of not administering adjuvant treatment and start follow-up.

The NGS panel test revealed only the already known mutation in *BRAF* c.1799T>A p.V600E. Although he had no family history to report and *BRAF* mutation was present, he had a germline testing performed with the finding of heterozygous p.G396D pathogenic mutation (also known as c.1187G>A) located in coding exon 13 of the *MUTYH* gene. This mutation is involved in a significantly reduced oxidative DNA mismatch repair efficiency though it has not been linked to a higher risk of colon cancer but of developing breast cancer (21–24). The hypermethylation of the *MLH1* promoter has not been performed.

## 3 Current state of the art, challenges, and future directions: discussion

Nowadays, immunotherapy is the SOC for irresectable and metastatic dMMR/MSI-H CRC; on the one hand, pembrolizumab, an antiPD-1 agent, is approved in 1**^st^** and further lines because of the remarkable results presented in the KEYNOTE 177 and KEYNOTE 016 vs. SOC chemotherapy (13, 25, 26). Besides, Nivolumab and the combination of Nivolumab plus Ipilimumab are also approved for the same settings as pembrolizumab due to the efficacy demonstrated in the CHECKMATE 142 phase III clinical trial (13, 27).

The role of immunotherapy in the neoadjuvant setting of dMMR/MSI-H CRC will be soon consolidated due to the remarkable results presented in recent studies: In the phase II study NICHE2, patients with locally advanced CRC were treated with one dose of ipilimumab and two doses of nivolumab and underwent surgery ≤6 weeks since starting therapy. An impressive mPR rate of 95%, including 67% pCR, was reported and the incorporation of immunotherapy as SOC at the neoadjuvant setting in these tumors is a matter of time (16). The phase II PICC trial randomized locally advanced dMMR/MSI-H CRC patients to received toripalimab, an antiPD-1, with or without celecoxib, for six cycles before surgical resection, with 88% of pCR in the celecoxib group (vs 65% in toripalimab monotherapy group) (19). The role of pembrolizumab in this setting has been evaluated in another phase II trial in which patients with localized unresectable or high-risk resectable dMMR/MSI-H solid tumors, including 27 CRC, were allocated to receive pembrolizumab for 6 months followed by surgical resection with an option to continue therapy for 1 year: a pCR was observed in 79% of CRC tumors operated (20). Recently, results from NICHE-3 were presented, which showed even better results in terms of pCR in the same setting of patients: after two cycles of nivolumab plus relatlimab, all the patients obtained a response, including 89% of mPR and 79% of pCR (18). The role of dostarlimab, another antiPD-1 checkpoint inhibitor, as neoadjuvant treatment in locally advanced rectal cancer also showed a remarkable 100% clinical complete response (cCR), allowing these patients to spare chemoradiotherapy and surgery, with no relapse evidence during follow up. These results have been confirmed in a 20-patient retrospective study: a 90% cCR was observed and pCR was confirmed in all the 11 patients that underwent surgery; the other 7 patients chose a watch-and-wait strategy, with no local or distant recurrence observed in neither of the groups (28). Interestingly, none of these trials are evaluating the role of immunotherapy as conversion therapy in oligometastatic disease and data from patients that could be elected for surgery in the metastatic trials published were censored. The principal clinical trials investigating the role of ICIs in the neoadjuvant setting and the results available are summarized in **Table 2**.

**Table 2:** Currently ongoing and completed clinical trials of ICI strategies in dMMR/MSI-H locoregional CRC.

Study	Treatment	Phase	Endpoint 1°	Setting	Cycles	Results	Status
**NCT05131919 (PUMA)** (29)	Pembrolizumab (PD-1 inhibitor)	II	ORR	Locally Advanced, Irresectable, Non-metastatic MSI-H/dMMR CRC	Maximum 2 years, or until the tumor becomes resectable.	NA	Active, recruiting
**NCT05239546 (NAIO)** (30)	Dostarlimab (PD-1 inhibitor)	II	MCR Rate at 18 weeks - avoid surgical resection	Stage II and III MSI-H/dMMR CRC	2 years	NA	Active, recruiting
**NCT04988191** (31)	Toripalimab (PD-1 inhibitor) + Bevacizumab + Irinotecan	Ib/II	% of pCR	T4a-b resectable MSI-H/dMMR CRC.	Toripalimab NA – 3 cycles Adjuvant – 9 cycles Irinotecan (2 cycles) +Bevacizumab (3 cycles) only in NA	NA	Active, recruiting
**NCT05815290** (32)	Cadonilimab (PD-1/CTLA-4 bi-specific antibody)	II	% of CR (pCR and cCR) - avoid surgical resection	MSI-H/dMMR locally advanced CRC	8 cycles	NA	Active, recruiting
**NCT05913570** (33)	Cadonilimab (PD-1/CTLA-4 bi-specific antibody)	II	% of pCR	Resectable Stage II-III MSI-H/dMMR CRC	4 cycles	NA	Active, not recruiting
**NCT05371197** (34)	Envafolimab (PD-L1 inhibitor)	II	% of pCR	Resectable Local Advanced dMMR/MSI-H CRC	4 cycles	NA	Active, recruiting
**NCT04715633** (35)	Camrelizumab + Apatinib If SD or PD – capecitabine + oxaliplatin as salvage therapy	II	% of CR (pCR and cCR) - avoid surgical resection	Resectable Local Advanced dMMR/MSI-H CRC	8 cycles	NA	Active, not recruiting
**NCT05841134** (36)	Tislelizumab combined with CAPOX – neoadjuvant and adjuvant therapy	II	% of CR (pCR and cCR) - avoid surgical resection	Stage II or III MSI-H/dMMR CRC	Neoadjuvant – 4 cycles Adjuvant – tislelizumab +/- CAPOX a maximum of 12 months	NA	Active, not recruiting
**NCT04556253** (37)	AK104 (PD-1/CTLA-4 bispecific antibody)	II	% of pCR	Stage II or III MSI-H/dMMR CRC	Not specified	NA	Active, not recruiting
**NCT05116085** (38)	Tislelizumab (PD-1 inhibitor)	II	% of MPR	Stage II or III MSI-H/dMMR CRC	Not specified	NA	Active, not recruiting
**NCT05197322 (NEOPRISM-CRC-)** (39)	Pembrolizumab	II	% of pCR	Stage II or III MSI-H/dMMR CRC	3 cycles if TMB high/medium 1 cycle if TMB low	NA	Active, recruiting
**NICHE (NCT03026140)** (17)	Ipilimumab/Nivolumab pMMR: +/- celecoxib	II	Safety and DFS	Stage III MSI-H/dMMR and MSS/pMMR CRC	1 cycle Ipi/Nivo + 1 cycle Nivo	dMMR - 100% (20/20) had pathological response - 95% (19/20) MPR (including 60% [12/20] pCR) pMMR - 4/15 (27%) PR (3 MPRs + 1 partial response)	Active, recruiting
**NICHE 2 (NCT03026140)** (16)	Ipilimumab/Nivolumab	II	Safety and DFS	Stage III MSI-H/dMMR CRC	1 cycle Ipi/Nivo + 1 cycle Nivo	93% MPR - 67% pCR.	Active, recruiting
**NICHE 3 (NCT03026140)** (18)	Nivolumab/Relatlimab	II	Safety and DFS	Stage III MSI-H/dMMR CRC	2 cycles Nivolumab/Relatlimab	89% MPR - 79% pCR	Active, recruiting
**PICC trial (NCT03926338)** (19)	Toripalimab +/- celecoxib	II	% of pCR	Stage III MSI-H/dMMR CRC and rectal cancer (n=4)	6 cycles Toripalimab: +/- celecoxib - +/- adjuvant treatment	88% (celecoxib group) vs 65% pCR	Active, recruiting
**NCT04082572** (20)	Pembrolizumab	II	Safety and pCR	Localized unresectable or high-risk resectable MSI-H/dMMR tumours (including CRC)	6 months +/- 1 year adjuvant pembrolizumab	27 CRC patients/35 - 17 had surgery. → 79% (11/14 CRC) had pCR. - Radiographic response → CR - 30% (10/33) → PR - 52% (17/33).	Active, not recruiting

MSI-H/dMMR CRC, high microsatellite instability-deficit of mismatch repair proteins colorectal cancer; ORR, overall response rate; MPR, major clinical response; pCR, Pathological complete response; cCR, clinical complete response; DFS, disease-free survival; NA, Not available.

- and → are for dividing the results from the trial (since not all of them had surgery and they also considered the radiographic response).

However, several questions are raised because of these studies and the ones coming: The necessity of using double immunotherapy instead of monotherapy, even if it is only one cycle, may be one of the issues to be assessed. Grade 3 or 4 adverse effects were only observed in 3 (3%) patients in the NICHE2 and 1 (5%) of patients in the NICHE3 trial. Curiously in the latter, 4 out of the 19 patients included experienced an endocrinopathy, including a grade 3 hyperthyroidism and 3 hypophysitis with secondary adrenal and/or thyroid disfunction that require a supplementation for a lifetime. Besides, a late adverse effect may appear, and the toxicity of double therapy is notably higher than the one observed with monotherapy. However, the necessity of response may be the argument used for this neoadjuvant unresectable setting (27, 40). In the metastatic scenario, the phase III Checkmate 8HW (NCT04008030) trial compares monotherapy (nivolumab) vs. double therapy (ipilimumab/nivolumab) (41), whose results may bring some light on this issue. Moreover, different doses of nivolumab plus ipilimumab are also being tested in dMMR/MSI-H mCRC in a phase II trial to analyze the efficacy and safety (NIPISAFE) (42). On the other hand, several trials are testing the percentage of mPR achieved with antiPD-1 monotherapy in the neoadjuvant setting in patients with locally advanced irresectable dMMR/MSI-H CRC (**Table 2**).

Another question to be addressed is the number of cycles necessary: in the NICHE2 trial, only two cycles (one combined and one monotherapy) were used before surgery, whereas other trials are proposing other numbers of cycles, an undetermined number of cycles for up to two years or until the tumour becomes resectable (16, 29, 30). The use of other dynamic biomarkers may be useful to determine the number of cycles or even the necessity of adjuvant therapy after surgery, such as ctDNA (43). A phase II trial is testing the utility of tumour mutation burden (TMB) as a biomarker of response to immunotherapy and if TMB is high (>20 mutations per Mb) or medium (>5 mutations per Mb) we can continue with pembrolizumab as neoadjuvant treatment for up to three cycles and if TMB is low (≤5 mutations per Mb) the patient can forgo pembrolizumab and proceed to surgery (39). Curiously, none of our cases had an elevated CEA despite the locoregional advanced setting or oligometastatic setting and, therefore, tumoral markers may not be trustworthy for making decisions or in the follow-up of these patients. Besides, there was no correlation either between the radiologic evolution and the pathological responses, even if remarkable responses were observed the mass was still visible; hence, if an organ-preservation strategy becomes an option in the future alternative modalities of re-staging may be superior and should be considered, such as PET-CT and/or a new colonoscopy to take biopsies (44).

Besides, the necessity of adjuvant therapy after applying neoadjuvant treatment also seems controversial; none of the patients of NICHE2 have had disease recurrence reported yet. However, this question may be more useful among the 5% that did not achieve a mPR but “only” partial response (4%) or no pathological response (1%) (16). In the KEYNOTE-177 trial, patients that could be elected for surgery were censored from the published data and, hence, we still have no data supporting or not supporting adjuvant therapy in these patients (25).

In previously untreated patients with stage II/III dMMR/MSI-H locally advanced rectal cancer, the use of dostarlimab, an antiPD-1 ICI, showed cCR and surgery could be avoided, with no relevant toxicity reported (no grade¾4 adverse effects) (45). This organ-preservative strategy is another question that should be tabled in dMMR/MSI-H locally advanced CRC, since some patients may benefit from disposing of surgery and some phase II trials are addressing this strategy either with monotherapy, such as dostarlimab (NCT05239546) or using a PD-1/CTLA-4 bispecific antibody (NCT05815290, NCT04556253) (30, 32, 37), with recent results that support this strategy following pembrolizumab (20).

The role of neoadjuvant treatment in oligometastatic disease, resectable or potentially resectable, has not been addressed in clinical trials either. Standard treatment includes 5-FU-based chemotherapy with or without monoclonal antibodies depending on the possibility of resection (3). However, as we have reported in our third and fourth clinical cases, these patients could benefit from using ICI due to the responses achieved and further investigation is required to establish its role either as monotherapy or the necessity of using double therapy if a response is needed. Although previous data on non-operable oligometastatic dMMR/MSI-H had shown deep and durable responses (46), no previous reports of oligometastatic dMMR/MSI-H CRC that underwent surgery after neoadjuvant ICI have been published and data from KEYNOTE-177 were censored from initial (25, 47).

Nowadays, a chemotherapy-sparing strategy seems reasonable for these patients and the use of combination strategies may have a place in selected cases (31) or if no response is achieved: a phase II study proposed a chemotherapy-salvage therapy when stable or progression disease is achieved after three cycles of Camrelizumab (antiPD-1) plus Apatinib (anti-VEGF) (35). The rates of pCR available from trials are remarkable, ranging from 60% (NICHE) to 88% (PICC), and almost reaching 100% when considering MPR (89-95%). Low rates of non-responder patients are reported (1% in NICHE2) (16, 17, 19), and patients that experienced “only” pathological response (50% or less residual viable tumour) are also scarce: 5% in NICHE, 4% in NICHE2 and 1% in PICC trial (corresponding to the combination arm) (16, 19). These data is striking when comparing to the data of progression disease as best response reported in metastatic trials (29,4% in KEYNOTE 177, 13% in CHECKMATE 142), although the substantially greater number of patients in the metastatic trials should be considered when analyzing these data (25, 48).

Therefore, biomarkers of response to ICIs are required in order to make an adequate selection of the patients that are candidate to neoadjuvant treatment, especially if a surgery option is available. Several retrospective studies have tried to elucidate clinical patterns of response to ICI: TMB^high^ (cut-point was estimated between 37 and 41 mutations/Mb) was predictive of response whereas the presence of peritoneal metastases and poor performance status were predictive of low benefit (13, 49). Focusing on the differences that these patients may have on their samples that may guide the response to ICIs, when using a multiplex immunofluorescence (MIF) staining and image analysis a higher pre-existing CD8+ T-cell density has been significantly associated with pCR response (767.47 per.mm2 vs. 326.64 per.mm2 [p= 0.013]) as well as a negative or low expression of PD-1 CD8+ cells (defined by an expression of *PDCD1* of 0; s (700.24 per.mm2 vs. 288.29 per.mm2, [p=0.007]). This associates with the already known absence of correlation of response with PD-L1 expression in the metastatic setting. The signature genes that were highly expressed in these PD-1^low^-CD8+ lymphocytes were *TRGC2*, *CD160*, and *KLRB1*, among others (50). On the other hand, the presence of Caudal-related homeobox transcription factor 2 (CDX-2) seem to be correlated with higher sensitivity to immunotherapy. This transcription factor is inversely associated with poor prognosis since it is downregulated by the oncogenic pathways activated in CRC. It promotes the expression of chemokine (C-X-C motif) ligand 14 (CXCL14) expression by activating its enhancer, promoting the migration and cytotoxicity of NK cells and, therefore, enhancing the immune response to ICIs. Although small (n=14) and performed in metastatic patients, this study showed a PFS rate at 12 months of 81% in CDX-2 positive patients (tested by IHC) vs 0% in CDX-2 negative patients (p = 0.0011) (51). Further studies are warranted to identify new biomarkers that could guide clinicians to choose the right therapy for these dMMR/MSI-H CRC patients.

Finally, the molecular characterization could also be useful in these patients: although *BRAF* mutation is classically connected with a worse prognosis, the possibility of using immunotherapy when dMMR/MSI-H status is associated has changed the scenario of these patients. Although one of our cases could not have an NGS test done due to lack of material, two of our cases had a *BRAF* V600E mutation using molecular testing, with no further alterations found in NGS and one of the wild-type cases had a *RET::NCOA4* fusion that could be targetable if needed in the future. When reviewing the literature, a higher proportion of mutations can be found even in the presence of *BRAF* mutations (52), and we can hypothesize that additional mutations may be accumulated throughout the evolution of the disease.

On the other hand, immunotherapy has not demonstrated yet its efficacy in advanced CRC patients that are microsatellite stable with proficient mismatch-repair proteins (pMMR/non-MSI-H) that represent most of CCR. However, an interesting overall response rate of 30% has been observed in this subgroup of patients in the NICHE1 clinical trial (**Table 2**), so these localized patients may be an interesting group on whom to investigate the reasons why they obtained these response rates and try combinational strategies (17). There is a need for trusty biomarkers of response to immunotherapy in this subgroup of patients, such as *POLE* mutation (53), as well as new therapeutic strategies that may allow these patients to benefit from the remarkable responses associated with ICIs (12).

## 4 Conclusions

This case series underlines the potential efficacy that pembrolizumab has in the neoadjuvant setting of the dMMR/MSI-H locoregional CRC, concurring with results from phase II neoadjuvant trials available. Nonetheless, our case reports also showed remarkable efficiency in oligometastatic disease, in which the use of ICIs as conversion therapy is still an unresolved topic. Several questions such as the best treatment scheme and duration, the necessity of adjuvant therapy or the establishment of organ-preservation strategies. are being questioned and, therefore, further investigation is warranted.

## Data availability statement

The original contributions presented in the study are included in the article/**Supplementary Material**. Further inquiries can be directed to the corresponding author.

## Ethics statement

Written informed consent was obtained from the individual(s) for the publication of any potentially identifiable images or data included in this article.

## Author contributions

MS-R-G: Conceptualization, Investigation, Visualization, Writing – original draft, Writing – review & editing, Methodology, Validation. IM-D: Investigation, Methodology, Resources, Supervision, Validation, Writing – review & editing. VA-F: Writing – review & editing. PG-S: Writing – review & editing. JP-P: Writing – review & editing. JC-P: Writing – review & editing. DR-R: Writing – review & editing. PS-R: Writing – review & editing. AB-C: Writing – review & editing. VA-N: Writing – review & editing. CG-M: Writing – review & editing. CG-D-Q-S: Writing – review & editing. VL: Resources, Writing – review & editing. IR-C: Resources, Writing – review & editing. CP-M: Resources, Writing – review & editing. RF-M: Investigation, Methodology, Resources, Supervision, Validation, Writing – review & editing.
