# Supplementary material for: Case report: Efficacy of immunotherapy as conversion therapy in dMMR/MSI-H colorectal cancer: a case series and review of the literature
Source: Front Immunol. 2024 Feb 1;15:1352262. doi: 10.3389/fimmu.2024.1352262 (PMC10867218; doi:10.3389/fimmu.2024.1352262)
Supplement: Supplementary file 1 [file DataSheet_1.docx]

Supplementary Material

# Supplementary Figures and Tables

## Supplementary Figures


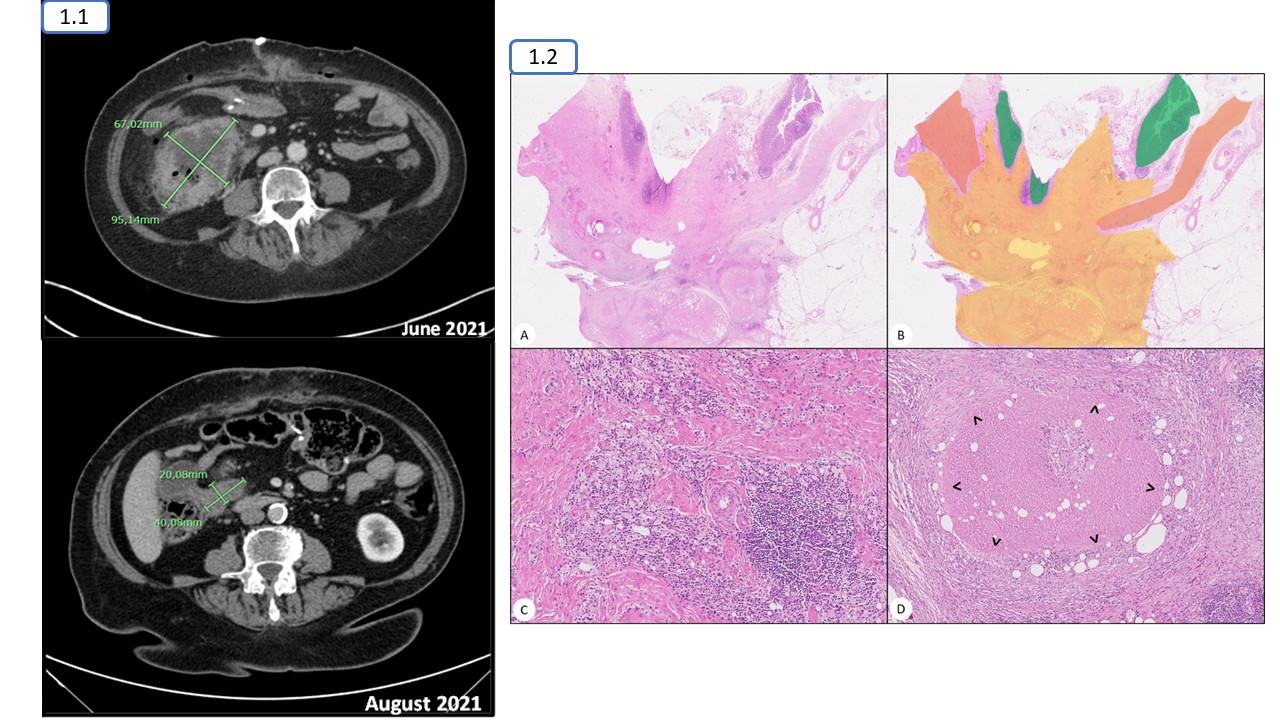


**Figure 1. 1.1.** The right colonic mass experienced a considerable decrease in its size from June to August 2021, with no longer evidence of liver or pancreatic infiltration **1.2.** (A, B) Transmural full-thickness section of the rectum showing regression changes with no residual neoplasia. Normal mucosa and muscularis propia are coloured green and orange, respectively. The area showing regression changes is coloured in yellow. (C) Some areas of regression showed a mixture of lymphoid and macrophage infiltrates admixed in a collagenous residual stroma. (D) Numerous necrotizing granulomas with a rim of macrophages (>) were present.


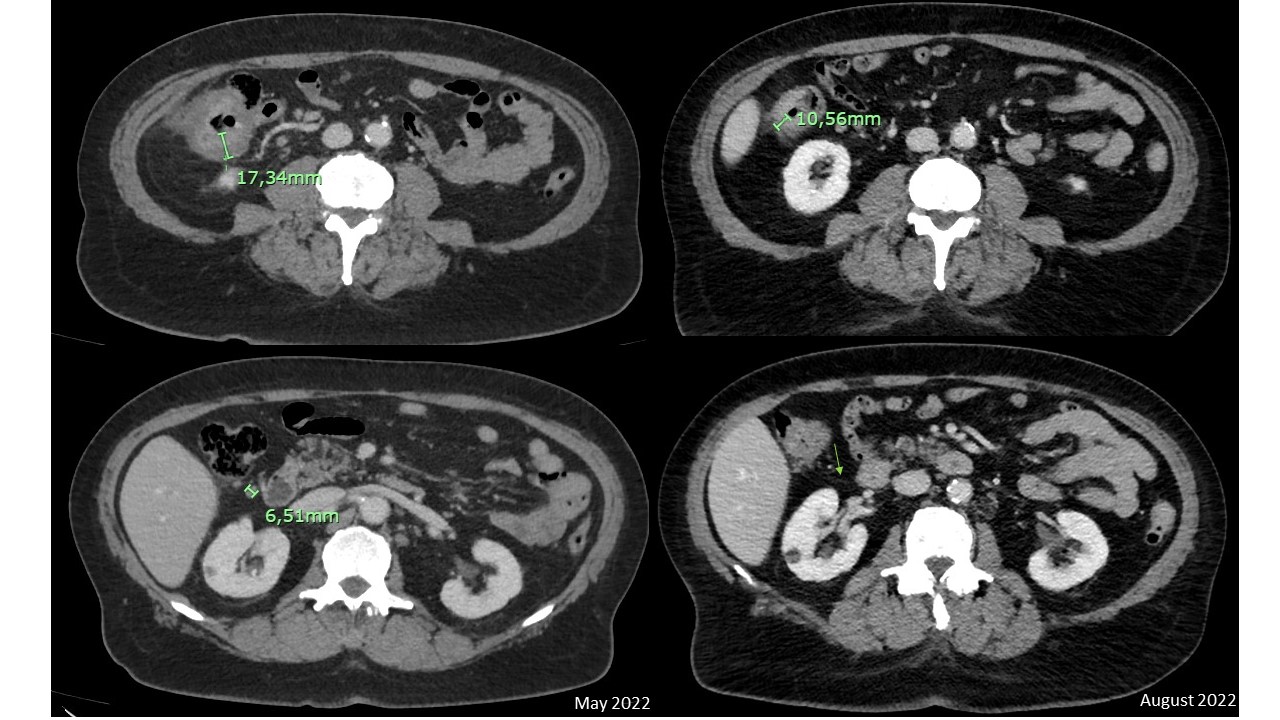


**Figure 2.** A major radiologic response was observed with a clear reduction of the colonic neoplasm and no longer evidence of adjacent suspicious nodes between the basal CT scan and interval CT scan in August 2022. The green arrow represents de absence of lymph node after 5 cycles of pembrolizumab.


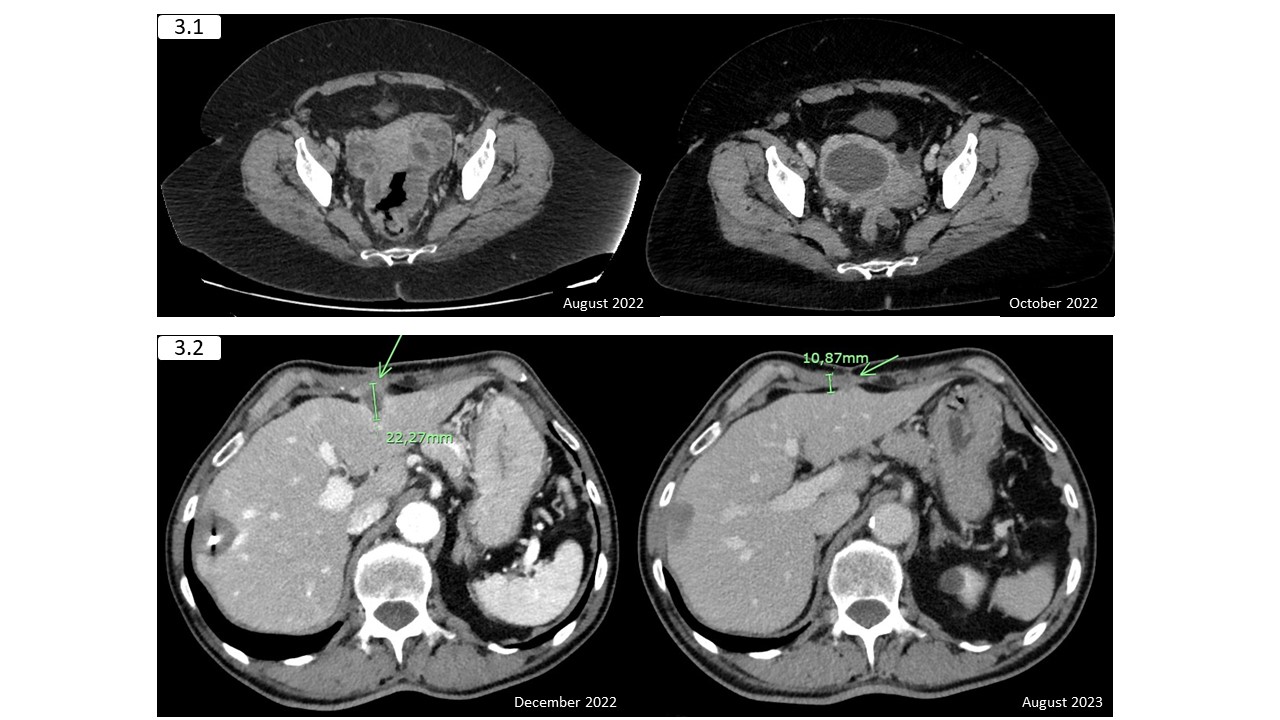


**Figure 3. 3.1 (Case report 3)** - The interval CT scan revealed an amelioration of the rectal and adnexal infiltration, with the apparition of a pyometra or a right-annex abscess that provokes an ipsilateral ureterohydronephrosis. A decrease in the abdominopelvic nodes was also observed. **3.2 (Case report 4)** - The previously treated with radiofrequency 33-millimetre liver lesion in the right hepatic lobe showed no changes and a remarkable decrease is appreciated in the segment III metastasis, remaining the signs of the hepatic capsule and anterior abdominal wall infiltration. The green arrow indicates the location of the infiltrating liver lesion.

## Supplementary Table 1

|  | Case Report 1 | Case Report 2 | Case Report 3 | Case Report 4 |
| --- | --- | --- | --- | --- |
| Family history  Tumour characteristics | Yes  MLH-1 and MSH-2 deficient poorly differentiated adenocarcinoma - Grade 3 | No  MLH-1 and PMS-2 deficient moderately differentiated adenocarcinoma - Grade 2 | No  MLH-1 and PMS-2 deficient adenocarcinoma with a component of signet ring cell -Grade 3 | No  MLH-1 and PMS-2 deficient well-differentiated adenocarcinoma -Grade 1 |
| Molecular findings^ | dMMR *RAS/BRAF* wild-type status (NGS not performed) | dMMR *BRAF* V600E/D (NGS performed) | dMMR *RAS/BRAF* wild-type status (NGS: *RET::NCOA4*) | dMMR *BRAF* V600E/D (NGS performed) |
| Staging pre-ICI | cT4N2Mx (pancreatic and liver infiltration) | cT3-T4N2M0 (Locally advanced unresectable) | cT4N2M1 (non-locoregional nodes and uterine and adnexal infiltration) | cTxNxM1 (liver relapse) |
| Number of cycles before surgery | 5 (pembrolizumab)* | 5 (pembrolizumab)* | 9 (pembrolizumab)* | 11 (pembrolizumab)* |
| Clinical Response | cT3N1-2Mx (no signs of pancreatic and segment IV liver infiltration) | cT2N1Mx (remarkable response of the primary mass and locoregional nodal disease) | cT3N1M1 | cTxNxM1 (liver relapse - major partial response) |
| Pathological Response | pCR | pCR | MPR [ypT3 ypN1a (1/27)] | pCR |
| Germline testing^ | Yes – sporadic (h*MLH1*) | No (*BRAF*m + h*MLH1*) | Yes – sporadic (h*MLH1*) | Yes – heterozygous p.G396D *MUTYH*^γ^ |
| Table 1: Clinical characteristics of the 4 case reports presented. ICI: immune-checkpoint inhibitors. pCR: Pathological Complete Response. MPR: Major Pathological Response. h*MLH1*: hypermethylation of *MLH1* promoter.  ^Molecular findings were obtained using a PCR method for KRAS and *NRAS-BRAF* mutation (Idylla™) and an NGS gene panel for further characterization (Oncomine™ Focus Assay), which enables the detection of variants in 52 genes relevant to solid tumours, such as relevant hotspots, single nucleotide variants (SNVs), indels, copy number variations (CNVs), and gene fusions. The dMMR status was determined in the first tissue available (endoscopic biopsy) by using an IHC test. *MLH1* promotor hypermethylation was assessed by using a methylation-specific PCR.  *Pembrolizumab posology was every three weeks in the neoadjuvant setting. Case Report 3 continued with adjuvant pembrolizumab every six weeks.  ^γ^This mutation has not been linked to a higher risk of CRC. | | | | |

## Supplementary Table 2

| Study | Treatment | Phase | Endpoint 1º | | Setting | Cycles | Results | Status |
| --- | --- | --- | --- | --- | --- | --- | --- | --- |
| NCT05131919 (PUMA) (32) | Pembrolizumab (PD-1 inhibitor) | II | | ORR | Locally Advanced, Irresectable, Non-metastatic MSI-H/dMMR CRC | Maximum 2 years, or until the tumour becomes resectable. | NA | Active, recruiting |
| NCT05239546 (NAIO) (33) | Dostarlimab (PD-1 inhibitor) | II | | MCR Rate at 18 weeks - avoid surgical resection | Stage II and III MSI-H/dMMR CRC | 2 years | NA | Active, recruiting |
| NCT04988191 (42) | Toripalimab (PD-1 inhibitor) + Bevacizumab + Irinotecan | Ib/II | | % of pCR | T4a-b resectable MSI-H/dMMR CRC. | Toripalimab  NA – 3 cycles  Adjuvant – 9 cycles  Irinotecan (2 cycles) +Bevacizumab (3 cycles) only in NA | NA | Active, recruiting |
| NCT05815290 (38) | Cadonilimab (PD-1/CTLA-4 bi-specific antibody) | II | | % of CR (pCR and cCR) - avoid surgical resection | MSI-H/dMMR locally advanced CRC | 8 cycles | NA | Active, recruiting |
| NCT05913570 (50) | Cadonilimab (PD-1/CTLA-4 bi-specific antibody) | II | | % of pCR | Resectable Stage II-III MSI-H/dMMR CRC | 4 cycles | NA | Active, not recruiting |
| NCT05371197 (51) | Envafolimab (PD-L1 inhibitor) | II | | % of pCR | Resectable Local Advanced dMMR/MSI-H CRC | 4 cycles | NA | Active, recruiting |
| NCT04715633 (43) | Camrelizumab + Apatinib  If SD or PD – capecitabine + oxaliplatin as salvage therapy | II | | % of CR (pCR and cCR) - avoid surgical resection | Resectable Local Advanced dMMR/MSI-H CRC | 8 cycles | NA | Active, not recruiting |
| NCT05841134 (52) | Tislelizumab combined with CAPOX – neoadjuvant and adjuvant therapy | II | | % of CR (pCR and cCR) - avoid surgical resection | Stage II or III MSI-H/dMMR CRC | Neoadjuvant – 4 cycles  Adjuvant – tislelizumab +/- CAPOX a maximum of 12 months | NA | Active, not recruiting |
| NCT04556253 (39) | AK104 (PD-1/CTLA-4 bispecific antibody) | II | | % of pCR | Stage II or III MSI-H/dMMR CRC | Not specified | NA | Active, not recruiting |
| NCT05116085 (53) | Tislelizumab (PD-1 inhibitor) | II | | % of MPR | Stage II or III MSI-H/dMMR CRC | Not specified | NA | Active, not recruiting |
| NCT05197322 (NEOPRISM-CRC-) (35) | Pembrolizumab | II | | % of pCR | Stage II or III MSI-H/dMMR CRC | 3 cycles if TMB high/medium  1 cycle if TMB low | NA | Active, recruiting |
| NICHE (NCT03026140) (18) | Ipilimumab/Nivolumab  pMMR: +/- celecoxib | II | | Safety and DFS | Stage III MSI-H/dMMR and MSS/pMMR CRC | 1 cycle Ipi/Nivo + 1 cycle Nivo | dMMR - 100% (20/20) had pathological response - 95% (19/20) MPR (including 60% [12/20] pCR)  pMMR - 4/15 (27%) PR (3 MPRs + 1 partial response) | Active, recruiting |
| NICHE 2 (NCT03026140) (17) | Ipilimumab/Nivolumab | II | | Safety and DFS | Stage III MSI-H/dMMR CRC | 1 cycle Ipi/Nivo + 1 cycle Nivo | 93% MPR - 67% pCR. | Active, recruiting |
| NICHE 3 (NCT03026140) (19) | Nivolumab/Relatlimab | II | | Safety and DFS | Stage III MSI-H/dMMR CRC | 2 cycles Nivolumab/Relatlimab | 89% MPR - 79% pCR | Active, recruiting |
| PICC trial (NCT03926338) (54) | Toripalimab +/- celecoxib | II | | % of pCR | Stage III MSI-H/dMMR CRC and rectal cancer (n=4) | 6 cycles Toripalimab: +/- celecoxib - +/- adjuvant treatment | 88% (celecoxib group) vs 65% pCR | Active, recruiting |
| NCT04082572 (21) | Pembrolizumab | II | | Safety and pCR | Localized unresectable or high-risk resectable MSI-H/dMMR tumours (including CRC) | 6 months +/- 1 year adjuvant pembrolizumab | 27 CRC patients/35   - 17 had surgery. - 79% (11/14 CRC) had pCR. - Radiographic response - CR - 30% (10/33) - PR - 52% (17/33). | Active, not recruiting |
| Table 2. Currently ongoing and completed clinical trials of ICI strategies in dMMR/MSI-H locoregional CRC. Abbreviations: MSI-H/dMMR CRC: high microsatellite instability-deficit of mismatch repair proteins colorectal cancer / ORR: overall response rate/ MPR: major clinical response / pCR: Pathological complete response / cCR: clinical complete response / DFS: disease-free survival / NA: Not available | | | | | | | | |
